# spreadr: An R package to simulate spreading activation in a network

**DOI:** 10.3758/s13428-018-1186-5

**Published:** 2019-02-20

**Authors:** Cynthia S. Q. Siew

**Affiliations:** 1 Department of Psychology, University of Warwick, Coventry, UK; 2 Department of Psychology, National University of Singapore, Singapore, Singapore

**Keywords:** Spreading activation, Network science, Computer simulation, Lexical retrieval, False memory, Clustering coefficient, Semantic priming, Semantic network

## Abstract

The notion of spreading activation is a central theme in the cognitive sciences; however, the tools for implementing spreading activation computationally are not as readily available. This article introduces the spreadr R package, which can implement spreading activation within a specified network structure. The algorithmic method implemented in the spreadr subroutines follows the approach described in Vitevitch, Ercal, and Adagarla (Frontiers in Psychology, 2, 369, 2011), who viewed activation as a fixed cognitive resource that could “spread” among connected nodes in a network. Three sets of simulations were conducted using the package. The first set of simulations successfully reproduced the results reported in Vitevitch et al. (Frontiers in Psychology, 2, 369, 2011), who showed that a simple mechanism of spreading activation could account for the clustering coefficient effect in spoken word recognition. The second set of simulations showed that the same mechanism could be extended to account for higher false alarm rates for low clustering coefficient words in a false memory task. The final set of simulations demonstrated how spreading activation could be applied to a semantic network to account for semantic priming effects. It is hoped that this package will encourage cognitive and language scientists to explicitly consider how the structures of cognitive systems such as the mental lexicon and semantic memory interact with the process of spreading activation.

Cognitive scientists view the mind as inherently associative, and an understanding of how the mind works necessitates an understanding of associative processing. A prominent theme in several theories of cognitive psychology is the idea of *spreading activation* (Anderson, 1983; Collins & Loftus, 1975), in which the activation of one concept in memory is thought to spread to, and activate, other closely related concepts. The notion of spreading activation has been invoked to account for a variety of cognitive phenomena, including semantic processing (Collins & Loftus, 1975; Collins & Quillian, 1969), semantic priming effects (Balota & Lorch, 1986; de Wit & Kinoshita, 2015), sentence processing (Traxler, Foss, Seely, Kaup, & Morris, 2000), errors in sentence production (Dell, 1986), false memories (Roediger, Balota, & Watson, 2001), and emotional influences on memory and processing (Bower & Cohen, 2014).

One implicit assumption of spreading activation that has curiously escaped discussion in the literature is that the spread of activation among concepts or words must necessarily occur within a given *cognitive structure* (e.g., long-term memory). This is an especially important point to consider, given the growing amount of research showing that the structure of cognitive systems affects processing in a variety of domains, including spoken word recognition (Chan & Vitevitch, 2009; Goldstein & Vitevitch, 2017), speech production (Chan & Vitevitch, 2010), visual word recognition (Siew, 2018; Yates, 2013), memory processes (Siew & Vitevitch, 2016; Vitevitch, Chan, & Roodenrys, 2012), semantic processing (Kenett, Levi, Anaki, & Faust, 2017), language acquisition in monolingual (Hills, Maouene, Maouene, Sheya, & Smith, 2009) and bilingual (Bilson, Yoshida, Tran, Woods, & Hills, 2015) children, word learning in adults (Goldstein & Vitevitch, 2014), and higher-order cognitive processes such as creativity (Kenett, Anaki, & Faust, 2014). In this body of research, cognitive systems are represented as a network with nodes and links connecting these nodes. For instance, a semantic network consists of nodes that represent individual words that are connected if they share a semantic relationship based on co-occurrences or free associations (De Deyne, Kenett, Anaki, Faust, & Navarro, 2016; Steyvers & Tenenbaum, 2005). Representing cognitive systems as networks permits the application of network science techniques in order to further examine the underlying structural properties of these cognitive networks (Baronchelli, Ferrer-i-Cancho, Pastor-Satorras, Chater, & Christiansen, 2013; Borge-Holthoefer & Arenas, 2010).

Given the prevalence of research articles discussing spreading activation as a key feature of cognitive theories and models (e.g., Anderson, 1983; Collins & Loftus, 1975), it is surprising that few computational tools to explicitly explore the notion of spreading activation exist; in addition, the tools that do exist do not tend to be specifically tailored for the needs of the psychologist. As was noted by Lewandowsky (1993), there are many benefits to conducting computer simulations to test out simple ideas about cognitive processes and to build stronger conceptual linkages between theory and behavioral data. This article fills this gap in the literature by introducing a computational tool for cognitive and language scientists who wish to conduct simulations of their own to examine spreading activation processes in their research area. That tool is the R package spreadr (pronounced “SPREAD-er”), which implements the spreading of activation among connected nodes (representing concepts or words) in a network (which could be viewed as an instantiation of semantic memory or the mental lexicon).

To investigate the theoretical concept of spreading activation, cognitive scientists have implemented models of random walks (and its variants) in the domain of semantic memory, in order to examine how people retrieve items from a category in fluency tasks (Abbott, Austerweil, & Griffiths, 2015), infer the structure of individual semantic networks from fluency data (Zemla & Austerweil, 2018), infer semantic similarity among words in a network of word associations (De Deyne, Navarro, Perfors, Brysbaert, & Storms, 2018), and examine search processes in people with high and low levels of creativity (Kenett & Austerweil, 2016). In its simplest implementation, a random walk is initiated from a specific node in the network, and the probability of moving from node *i* to node *j* is given by its transitional probability, as computed by:

$$ {T}_{ij}=\frac{A_{ij}}{\sum_{k=1}^n{A}_{kj}}, $$

where *A*_*ij*_ is the adjacency matrix of the network representation, whereby the presence of an edge between any two nodes in the network is indicated by a value of 1 (and 0 if the edge does not exist). The “walk” is permitted to continue for a certain number of steps, as specified by the modeler. When a large enough number of random walks have been implemented, researchers typically compute the probability that node *i* has been visited by the random “walker” or the probability that node *i* represents the final end point of the random walk; these probabilities are argued to reflect the “long-run” activation levels of nodes that would be produced by a spreading activation process implemented on the same network structure (Kenett & Austerweil, 2016).

Although random walk and spreading activation models lead to similar outputs (see Appendix 3 for simulations that demonstrate this), spreadr represents an important tool that complements random walk models and provides greater flexibility to the modeler. For instance, instead of conducting hundreds of thousands of random walks and compiling the results, the spreading activation process implemented in spreadr produces outputs that reflect the long-run behavior of random walks, leading to substantial savings of computational time (see Appendix 3). The spreadr package also includes a number of parameters (discussed below) that allow activation to decay over time or that increase or decrease the amount of activation spread to other nodes, which may be less straightforward to implement in the basic version of the random walk model. Although it should be emphasized that the present article does not aim to present a complete, formal theory of spreading activation and compare it against random walk models, spreadr does provide the tools that will enable future researchers to formalize and test models of spreading activation and compare them against random walk models and their variants.

At this point, it is also important to briefly acknowledge that other tools that can conduct simulations of diffusion processes in networks (which are analogous to the notion of spreading activation) do exist. Within the network science literature, there has been a strong interest in examining diffusion processes in network structures, and many open-source tools exist for researchers who study how epidemics and ideas might spread in social networks (e.g., the netdiffuseR package; Valente, Dyal, Chu, Wipfli, & Fujimoto, 2015). However, it is important to note that these tools model network diffusion in ways that specifically mimic the diffusion of a discrete event such as an epidemic. Specifically, an initial set of nodes is first “infected,” and the aim is to determine the proportion of nodes that adopt some type of discrete event (e.g., a disease) as a function of the overall network structure and parameters such as the probability of a connected node adopting the event (i.e., becoming infected). Although it is certainly possible to repurpose the notion of spreading activation as a diffusion process that “infects” certain nodes in a cognitive network with some amount of activation, this is arguably tedious and unnecessary, given that spreadr implements spreading activation in a way that is consistent with how spreading activation is commonly discussed and used in the cognitive sciences—where activation is viewed as a limited cognitive resource that can spread and activate connected words and concepts in long-term memory (Collins & Loftus, 1975). The spreadr package is designed to be highly accessible to psychologists and for addressing questions that psychologists are deeply interested in, such as language processing and memory retrieval.

Finally, it is important to emphasize that the network representation on which the spreading of activation process is being implemented is *not* a neural network. The term “neural network” comes from the connectionist framework and refers to a representation that consists of processing units that are connected to each other via weights acquired via a learning phase, and specific concepts are represented as distributed activity patterns in that representation. In contrast, the network representation discussed in the present context is “localist” in nature, whereby each concept/word is represented by a distinct node, and the modeler is required to explicitly define the relationships (or edges) that exist between nodes in the network representation.

As we shall see below, spreadr explicitly implements spreading activation in a *network* of interconnected nodes. This is a deliberate feature of spreadr, emphasizing a central tenet in the field of network science—that is, a complete understanding of any process we wish to investigate is not possible without a careful consideration of the *structure* within which those processes occur (Borge-Holthoefer & Arenas, 2010; Strogatz, 2001). Unlike the connectionist framework, the network science approach compels the modeler to be explicit about the edges and connections that give rise to the overall structure of the network representation, thereby allowing the researcher to deliberately study how specific structural properties of the system interact with the processes that occur in that system. Hence, spreadr represents an invitation to all researchers to explicitly study the interaction between structure and process in the cognitive and language sciences.

Especially germane to the present article is the set of computer simulations conducted by Vitevitch, Ercal, and Adagarla (2011) to investigate a possible account for the clustering coefficient effect observed in spoken word recognition. Vitevitch and colleagues (Chan & Vitevitch, 2009, 2010) found that across a variety of tasks, words with low clustering coefficients were processed more quickly than words with high clustering coefficients.

In the phonological network, nodes represent lexical representations, and edges are placed between words that are phonologically similar to each other (Vitevitch, 2008). Words that differ by the substitution, deletion, or addition of one phoneme are considered to be phonologically similar (Luce & Pisoni, 1998). Using the tools of network science, one can compute various similarity measures, such as *degree* and *clustering coefficient*. *Degree* represents the number of connections a node has. In the context of the phonological language network, degree is equivalent to the number of phonological neighbors that a word has in terms of the one-phoneme edit distance metric (i.e., phonological neighborhood density; Luce & Pisoni, 1998). For instance, the phonological neighbors of the word *cat* /kæt/ include *bat* /**b**æt/ (substitution), *at* /**_**æt/ (deletion), and *cast* /kæ**s**t/ (addition). *Clustering coefficient* represents the extent to which a word’s neighbors are also neighbors of each other (Watts & Strogatz, 1998). Clustering coefficient is computed using the following equation:

$$ {C}_i=\frac{2\left|{e}_{jk}\right|}{k_i\left({k}_i-1\right)}, $$

where *e*_*jk*_ refers to the presence of a connection between two neighbors *j* and *k*, and *k*_*i*_ refers to the degree (i.e., neighborhood density) of node *i*. Thus, the clustering coefficient represents the number of links that exist in a word’s neighborhood divided by the maximum number of links that could possibly exist in a word’s neighborhood. *C*_*i*_ ranges from 0 to 1, such that words with low clustering coefficients have a low level of connectivity among their neighbors (see the right side of Fig. 1), whereas words with high clustering coefficients have a high level of connectivity among their neighbors (see the left side of Fig. 1).

**Fig. 1:**
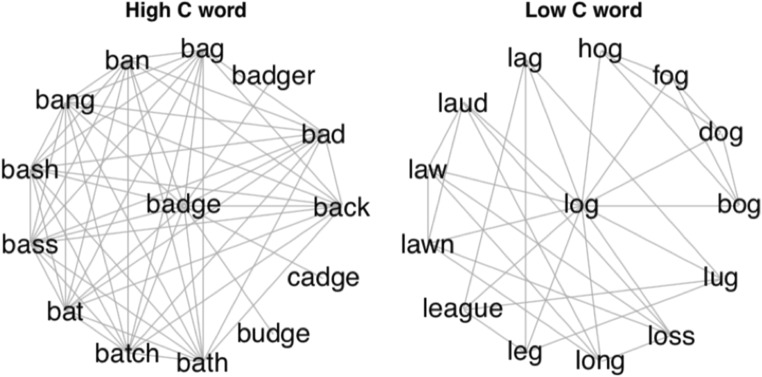
A word with a high clustering coefficient (*badge*; left) and a word with a low clustering coefficient (*log*; right). Note that although both words have the same degree (i.e., the same number of phonological neighbors), the internal connectivity of their phonological neighborhoods is very different

Given that current models of spoken word recognition were unable to account for the finding that words with low clustering coefficients were processed more quickly than words with high clustering coefficients, Chan and Vitevitch (2009) provided a post-hoc explanation of their findings. Beginning with the assumption that activation is a fixed cognitive resource that can “spread” among connected nodes in a network, Chan and Vitevitch (2009) suggested that for words with lower levels of interconnectivity, activation among the neighbors would spread back to the target word, with the remaining activation dispersing to the rest of the network (right side of Fig. 1). The target low *C* word would be strongly activated, resulting in rapid retrieval from the lexicon. On the other hand, for words with higher levels of interconnectivity, activation would likely remain among the interconnected neighbors rather than spread back to the target word or disperse to the rest of the network (left side of Fig. 1). This would lead the target high *C* words to be less strongly activated, resulting in less rapid retrieval from the lexicon.

Vitevitch et al. (2011) explicitly tested this verbal account in a computer simulation in which activation was allowed to spread among the words in a phonological network. In Vitevitch et al.’s (2011) implementation (whose algorithm was adopted in spreadr), the target node was assigned an arbitrary amount of activation. Some of that activation was retained by the node, and the rest was spread equally among the node’s neighbors. In the next time step, the same process was repeated for all nodes with nonzero activation levels. Vitevitch et al. (2011) allowed this process to be repeated ten times and then compared the final activation levels of words with high and low clustering coefficients. Higher levels of final activation indicated greater efficiency of lexical retrieval. Words with low clustering coefficients had higher final activation levels than words with high clustering coefficients, providing support for Chan and Vitevitch’s (2009) verbal account of their behavioral finding.

The simulations conducted by Vitevitch et al. (2011) may appear simplistic, but they provide important insights into how a simple process of spreading activation can lead to different outcomes, depending on the *structure* in which this process was operating. The development of spreadr was motivated by similar principles of parsimony, and its functions were designed to be as simple as possible, to enable generalizations and extensions to a broad range of investigations. In the following section, I first describe how the functions in spreadr were constructed, and provide a simple example to walk the user through its use. In the next section, the results of three simulation studies are reported. The first set of simulations demonstrated that the results described in Vitevitch et al. (2011) can be replicated using the spreadr package. The second set of simulations examined the clustering coefficient effect on false memory (Vitevitch et al., 2012), to demonstrate the utility of spreadr for investigating spreading activation in cognitive phenomena other than lexical retrieval. The final set of simulations was conducted on a *semantic* network, to further demonstrate the generality and usefulness of spreadr and how it can be used to investigate semantic priming.

## Implementation of spreading activation in a network

The functions created in the spreadr R package were written to implement the spreading activation process described in Vitevitch et al. (2011). The algorithmic details are provided below.

At each time step *t* and for each node *n* that has a nonzero activation value at *t* [i.e., *inflow*(*t*, *n*) > 0]:

(i) A proportion of activation is retained in node *n*, as given by the following equation:

$$ reservoir\left(t,n\right)=r\times inflow\left(t,n\right), $$

(ii) The nonretained activation is equally “spread” to all immediate neighbors of node *n*, as given by the following equation:

$$ outflow\left(t,n\right)=\frac{\left(1-r\right)\times inflow\left(t,n\right)}{\mathit{\deg}(n)}, $$

In addition, for all nodes in the network at each time step *t*:

(iii) The activation received from each of its neighbors are added to its own retained activation from the previous time step, as given by the following equation:

$$ inflow\left(t,n\right)=\sum \limits_{i=1}^{\deg (n)} outflow\left(t-1,{d}_i\right)+ reservoir\left(t-1,n\right), $$

where *reservoir* (*t*, *n*) is the amount of activation retained at node *n* at time step *t*, *inflow * (*t*, *n*) is the total amount of activation flowing into node *n* at time step *t*, *outflow* (*t*, *n*) is the activation flowing out of node *n* to each of its neighbors at time step *t*, *r* is the proportion of activation retained at node *n*, *d* is a neighbor of node *n*, and deg(*n*) is the number of neighbors of node *n.*

Calling the *spreadr* function in spreadr will invoke a subroutine that algorithmically implements actions (i)–(iii) for all nodes in the specified network for a given number of times.

### Parameters

The *spreadr* function includes a number of parameters that can be specified by the user. A detailed description of these parameters is provided below.

- *start_run*: This parameter takes the form of a data frame that contains the activation values assigned to specific nodes at *t* = 0.
- *retention*, *r*: This parameter refers to the proportion of activation that is retained by the node at each time step of the simulation. This parameter was manipulated by Vitevitch et al. (2011) to demonstrate that the simulation results were consistent across various values of retained activation; however, the retention parameter could also be manipulated in order to examine hypotheses related to the notion of an “increased” spread of activation (i.e., implemented as a lower proportion of activation retained) as a possible mechanism underlying hyperpriming effects observed in people with schizophrenia (Moritz et al., 2001; Pomarol-Clotet, Oh, Laws, & McKenna, 2008).
- *decay*, *d*: This parameter refers to the proportion of activation that is “lost” at each time step of the simulation. Although Vitevitch et al. (2011) did not manipulate *d*, thereby ensuring that the total sum of activation values in the network would remain constant over time, *d* was included as a parameter in the function in order to allow the researcher to reexamine the assumption that activation is a fixed cognitive resource that does not diminish over time, in line with previous empirical work suggesting that activation is a resource that can decay over time (Lorch, 1982; McKoon & Ratcliff, 1992).
- *suppress*, *s*: This parameter refers to the minimum activation value, whereby nodes with activations less than this minimum value at the end of each time step will have their activations “suppressed” to 0. The purpose of including this parameter is to speed up the simulations and instantiate the assumption that nodes with extremely low activation levels are essentially nonactive during the spreading activation process.
- *time steps*, *t*: This parameter refers to the number of time steps over which the spreading activation process occurs. Vitevitch et al. (2011) allowed activation to spread for ten time steps and assumed that lexical retrieval occurred at the end of ten time steps. The final activation levels of the target nodes were assumed to be positively correlated with processing efficiency (i.e., faster reaction times [RTs] or higher accuracy). It is important to note that, although there are different ways to implement the retrieval process, the different mechanisms commonly employed in those models (e.g., an activation threshold that must be crossed, different resting levels of activation, etc.) typically produce isomorphic results (McClelland & Rumelhart, 1981; Morton, 1969).

Finally, it is important to note that the selection of values for these parameters is somewhat arbitrary. However, many computational models typically include a large number of free parameters (e.g., McClelland & Elman, 1986), and what is most crucial is to ensure that the qualitative behavior of the model is robust under a range of parameter values. In computational work it is important for the researcher to be transparent about the values of the parameters that were tested in the simulation, enabling a thorough examination of the ability of the model to reproduce behavioral patterns.

## Step-by-step guide to using spreadr

The spreadr R package can be downloaded directly from the Comprehensive R Archive Network. The latest version of the package can also be downloaded from the author’s Github page. The source code for the functions used in spreadr can also be downloaded directly from the following website, https://github.com/csqsiew/spreadr, and researchers are welcome to download and modify the functions for their own purposes.

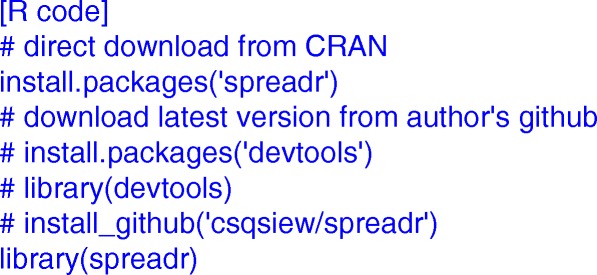



**Fig. 2:**
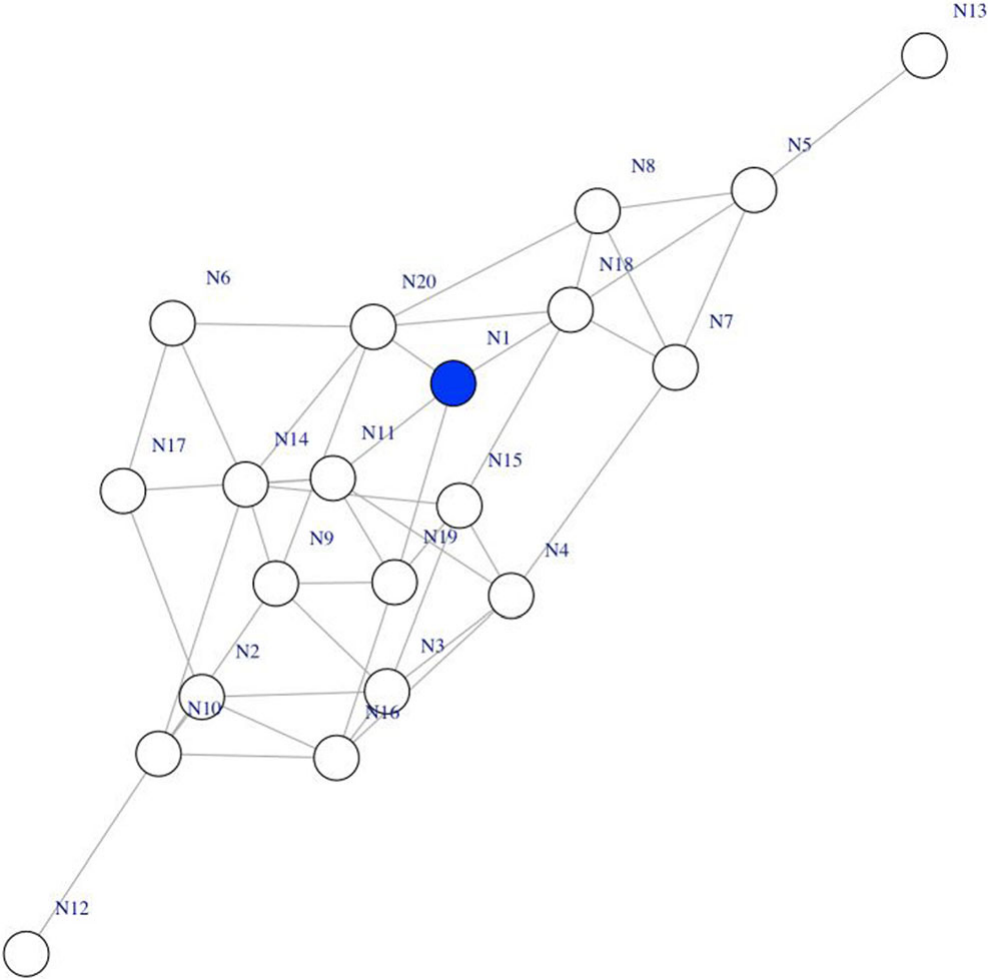
The randomly generated network in which the simulation was conducted. The blue node “N1” was initialized with 20 units of activation, some of which spread to nodes “N5,” “N8,” “N9,” and “N18” in the next time step

First, the network in which the spreading of activation occurs must be specified. In this example, we use the *sample_gnp* function from the igraph R package to generate a network with 20 nodes, and undirected links are randomly placed between pairs of nodes with a probability of .2 (Fig. 2). It is possible for the user to create a network from an edge list or an adjacency matrix. In this step, it is important to create a network object that is (i) recognized by igraph as a network object and (ii) has a meaningful *name* attribute (to specify the node labels). In addition, spreadr is able to conduct the simulation directly on an adjacency matrix without requiring conversion to an igraph object. Note also that the present network specified consists of unweighted, undirected links; however, it is possible to conduct simulations on networks with weighted and directed edges (see the detailed vignette provided at https://github.com/csqsiew/spreadr for more information about these advanced topics).

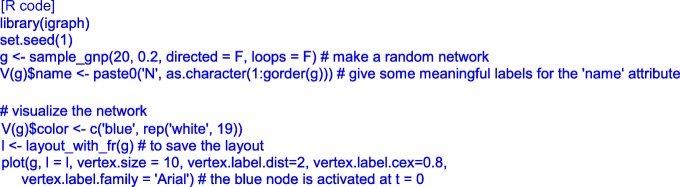



The user must then specify the initial activation level(s) of the node(s) in the network in a data frame object with two columns, labeled *node* and *activation*. Below the node labeled “N1” was assigned 20 units of activation. The user can choose to provide different activation values or to initialize more nodes with various activation values (a concrete example of this will be provided in Study 2 below).
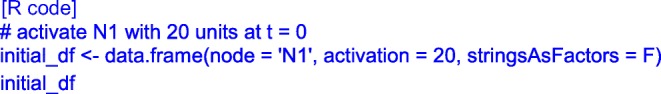


We are finally ready to run the simulation. In this step, the user must specify the following arguments and parameters in the *spreadr* function:

(i) *start_run*: the data frame (*initial_df*) specified in the previous step that contains the activation values assigned to nodes at *t* = 0;
(ii) *decay*, *d*: the proportion of activation lost at each time step (ranges from 0 to 1);
(iii) *retention*, *r*: the proportion of activation retained in the originator node (ranges from 0 to 1);
(iv) *suppress*, *s*: nodes with activation values lower than this value will have their activations forced to 0. Typically this will be a very small value (e.g., < .001);
(v) *network*: the network (N.B. must be an igraph object or an adjacency matrix) in which the spreading of activation occurs;
(vi) *time*, *t*: the number of times to run the spreading activation process, and
(vii) create_names: the default is TRUE, so that unique numeric labels will be created for the nodes in case they were not named in the network object.
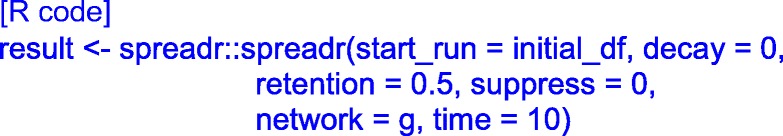

**Fig. 3:**
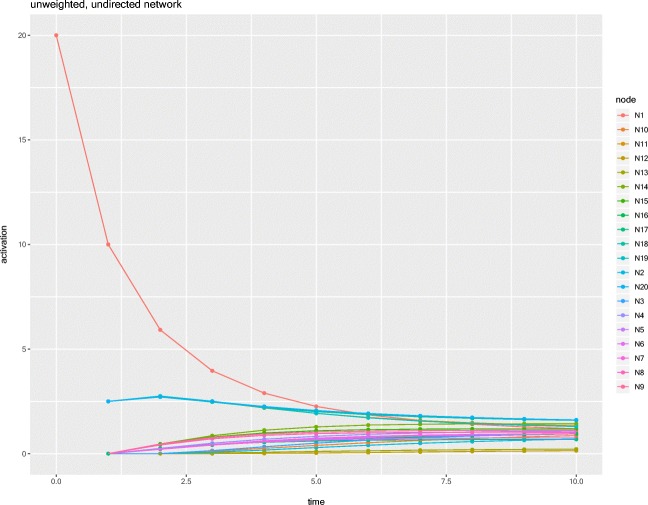
Results of the example simulation, where the activation values of all nodes in the network at each time step are plotted on the graph

The output is a data frame with three columns, labeled *node*, *activation*, and *time*, and contains the activation value of each node at each time step of the simulation. The output can easily be saved as a .csv file for further analysis later. A plot showing the activation levels of each node in the network at each time step is shown in Fig. 3.

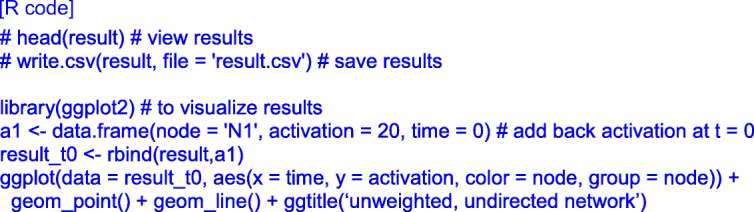



In the next section of this article, the results of three sets of simulations are reported. The first set of simulations serves as an “sanity check,” to ensure that spreadr is able to reproduce the results reported in Vitevitch et al. (2011) regarding the clustering coefficient effect in lexical retrieval. The second and third sets of simulations demonstrate how spreadr can be extended to investigate other aspects of cognitive phenomena, specifically the behavioral findings in false memory and semantic priming.

## Simulation Study 1: Lexical retrieval

The goal of Study 1 was to demonstrate that the implementation of the spreading activation process in spreadr is able to reproduce the results of Vitevitch et al. (2011). Hence, the steps taken followed the method described in Vitevitch et al. (2011) as closely as possible.

A total of 12 words with high clustering coefficients and 12 words with low clustering coefficients were selected from the phonological language network described in Vitevitch (2008). The 24 words were selected such that their degree, clustering coefficient, and two-hop network density (a measure indicating the overall connectivity of the target’s two-hop network) values were as closely matched as possible to the values listed in Appendix 1 of Vitevitch et al. (2011), who selected words across a wide range of degree values (3 to 40). The two-hop network consisted of the target node, its immediate neighbors, and the neighbors of its immediate neighbors (i.e., its two-hop neighbors). The network statistics of the 24 words used in the present set of simulations are provided in Appendix 1.

**Fig. 4:**
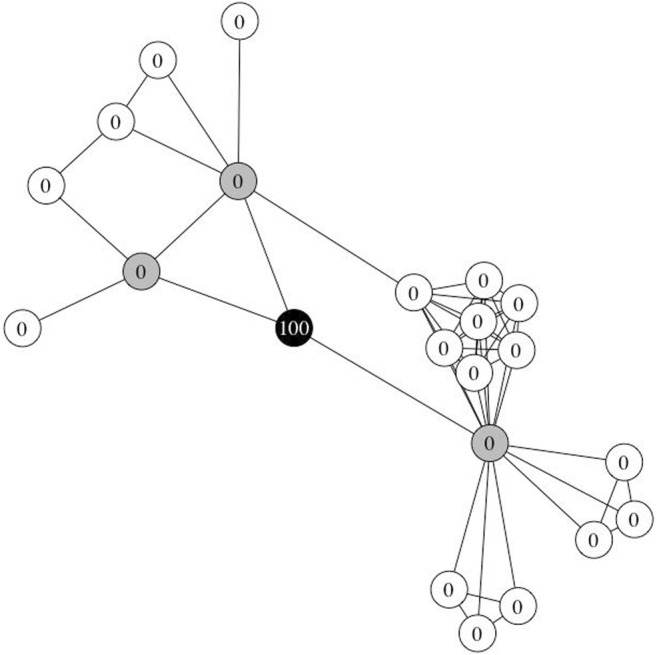
One of the 24 word networks used in Simulation Study 1. The target node is in black and was initially assigned 100 units of activation. The immediate (one-hop) neighbors of the target node are in gray, and the two-hop neighbors of the target node are in white. None of the one-hop or two-hop neighbors were assigned any activation at *t* = 0

The two-hop network for each of the 24 words was extracted and converted to an igraph network object for the simulations. In accordance with Vitevitch et al. (2011), the following parameters were used: nine different *retention* values (.1 to .9, in increments of .1), to ensure that the results would be generalizable across different parameter settings, and 100 units of activation were assigned to the target node at *t* = 0 (see Fig. 4). The *suppress* and *decay* parameters were set to 0, since Vitevitch et al. (2011) did not manipulate these parameters. A total of 216 simulations were conducted (24 word networks * 9 retention levels).

A linear regression model was conducted to examine the influences of degree, clustering coefficient, and retention on the activation values of the target node at the final time step. Note that the activation value of the target node at the final time step was taken to be a proxy for the efficiency with which the word was retrieved from the lexicon. Specifically, higher activations correspond to faster RTs and higher accuracy rates in psycholinguistic tasks. The predictors were retention, degree, and clustering coefficient, which were all included in the model as continuous variables.

**Fig. 5:**
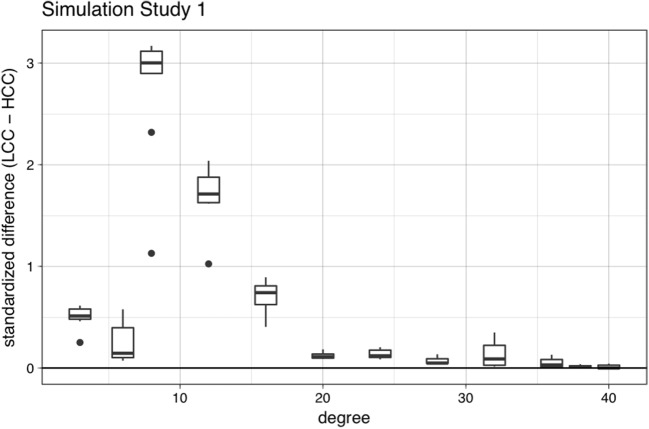
Mean standardized difference scores (with error bars) between the final activation of words with low clustering coefficients and words with high clustering coefficients with the same degree in Simulation Study 1. The difference score was obtained by subtracting the final activation of the high *C* word from the final activation of the low *C* word with the *same degree* and for the *same retention parameter* (i.e., for each value of degree, nine difference scores represented the nine different retention values [.1 to .9, in increments of .1]). The mean difference score was obtained by averaging across all retention values. In almost all cases, the final activation values of words with low clustering coefficients were greater than the final activation value of words with high clustering coefficients

Table 1 shows the standardized beta coefficients for each predictor and their corresponding *t* tests. The overall adjusted *R*^2^ was .550, *F*(3, 212) = 88.7, *p* < .001. Unsurprisingly, retention was a significant predictor of the final activation values, such that higher retention rates were associated with higher final activation values. Clustering coefficient was also a significant predictor of the final activation values, such that words with high clustering coefficients had lower final activations (corresponding to lower accuracy and slower RTs), replicating the simulations reported in Vitevitch et al. (2011), and consistent with the behavioral findings of Chan and Vitevitch (2009). Finally, degree was a significant predictor of the final activation values, such that words with high degrees had lower final activations (corresponding to lower accuracy and slower RTs), consistent with prior work in spoken word recognition regarding phonological neighborhood density effects (Luce & Pisoni, 1998) and mirroring the effect also reported in the original simulation (Vitevitch et al., 2011). Figure 5 shows the standardized difference scores between the activation levels of words with low clustering coefficients and words with high clustering coefficients across various values of degree. For almost all values of degree, the difference scores were positive, indicating that words with low clustering coefficients had higher final activations than words with high clustering coefficients, although the difference was larger for words with lower degrees (i.e., fewer immediate neighbors), and much smaller for words with higher degrees (i.e., many immediate neighbors), suggesting that the internal connectivity of a word’s neighborhood might have a greater influence on processing when a word’s neighborhood is smaller. The simulations suggest an interesting interaction between degree and clustering coefficient that could be examined in future empirical work.

**Table 1:** Standardized beta coefficients for each predictor in the regression model in Simulation Study 1, and their corresponding *t* tests

	*β*	*SE*	*t*	*p*
Retention	7.97	0.507	15.71	< .001
Degree	– 2.07	0.523	– 3.96	< .001
Clustering coefficient	– 1.48	0.520	– 2.83	.005

## Simulation Study 2: False memory

The goal of Study 2 was to demonstrate that the implementation of the spreading activation process in spreadr can be applied to investigate other aspects of cognitive processing—specifically, the emergence of false memories. In the original paradigm, participants studied a word list consisting of items semantically associated with the critical word *sleep*, but never the word *sleep* itself (e.g., *dream*, *bed*, *tired*, *awake*). In the test phase, during which participants recalled items from the study phase, the word *sleep* tended to be falsely recalled, even though it had not been presented during the study phase (Deese, 1959; Roediger & McDermott, 1995). Since then, others have adopted the same paradigm to investigate phonological false memory, in which lists of phonologically similar words were presented to participants during the study phase, and the participants tended to falsely recall words that sounded similar to those in the presented list (Sommers & Huff, 2003; Sommers & Lewis, 1999; Watson, Balota, & Sergent-Marshall, 2001).

For this set of simulations, we focused on the findings in Experiment 1 of Vitevitch et al. (2012), who investigated *phonological* false memory (e.g., Sommers & Lewis, 1999). In this study, Vitevitch et al. (2012) presented the phonological neighbors of words with high and low clustering coefficients during the study phase, but not the critical words themselves (i.e., the words with high and low clustering coefficients), and found that words with low clustering coefficients were more likely to be falsely remembered. This finding suggested that the internal connectivity of a word’s phonological neighbors might play a role in modulating the partial activation of the non-presented word (i.e., the critical word). In the present simulations, we sought to see whether these behavioral findings could be accounted for via the same process of spreading activation.

**Fig. 6:**
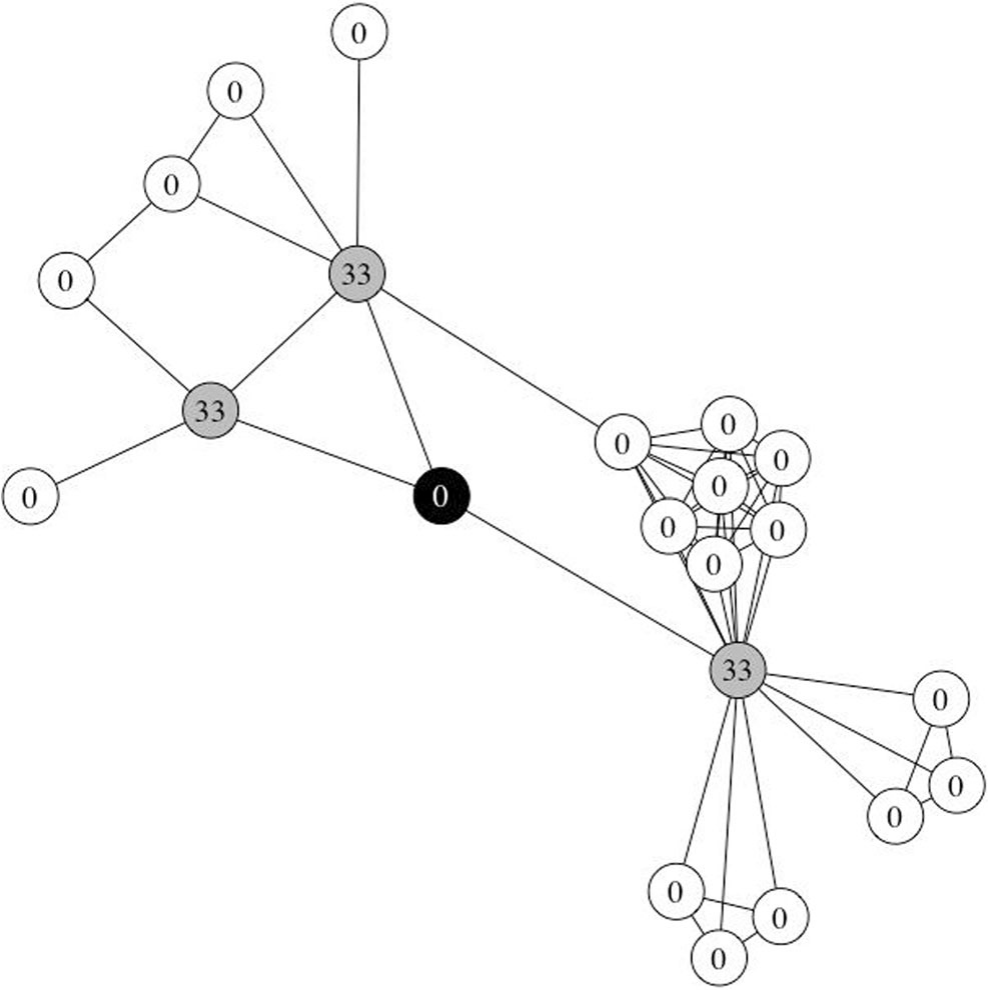
One of the 24 word networks used in Simulation Study 2. The target node is in black. The immediate (one-hop) neighbors of the target node are in gray and are assigned 33 units (= 100/3) of activation at *t* = 0. The two-hop neighbors of the target node are in white. None of the two-hop neighbors or the target node was assigned any activation at *t* = 0

The same two-hop networks for each of the 24 words from Study 1 were used in this set of simulations. Although these were not the same words used in the phonological false memory experiment conducted in Vitevitch et al. (2012), this set of 24 words from the earlier simulation was used because these words had already been selected to represent a wide range of degree and clustering coefficient values (i.e., 12 pairs of words with degrees ranging from 3 to 40, such that each pair had the same degree and contained a word with high *C* and another word with low *C*). The following parameters were used as before: nine different *retention* values (.1 to .9, in increments of .1), to ensure that the results would be generalizable across different parameter settings, and *suppress* and *decay* parameters set to 0. Instead of assigning activation to one node at the outset, activation was assigned to the target’s immediate neighbors. To ensure that the amount of activation that each network was initialized with was constant across all simulations, the amount of activation assigned to each neighbor of target node *i* was 100 units/degree of node *i*. Note that no activation was assigned to the target node, mirroring the false memory paradigm, in which a word’s semantic or phonological neighbors (but not the word itself) were presented during the study phase (Roediger & McDermott, 1995; see Fig. 6). A total of 216 simulations were conducted (24 word networks * 9 retention levels).

A linear regression model was constructed to examine the influence of degree, clustering coefficient, and retention on the activation value of the target node at the final time step. Note that the activation value of the target node at the final time step was taken to be a proxy for false alarm rates in false memory tasks. Specifically, higher activations correspond to a higher likelihood of false alarms (recognition or recall of a non-presented word) in false memory paradigms. The predictors were retention, degree, and clustering coefficient, which were all included in the model as continuous variables.

**Fig. 7:**
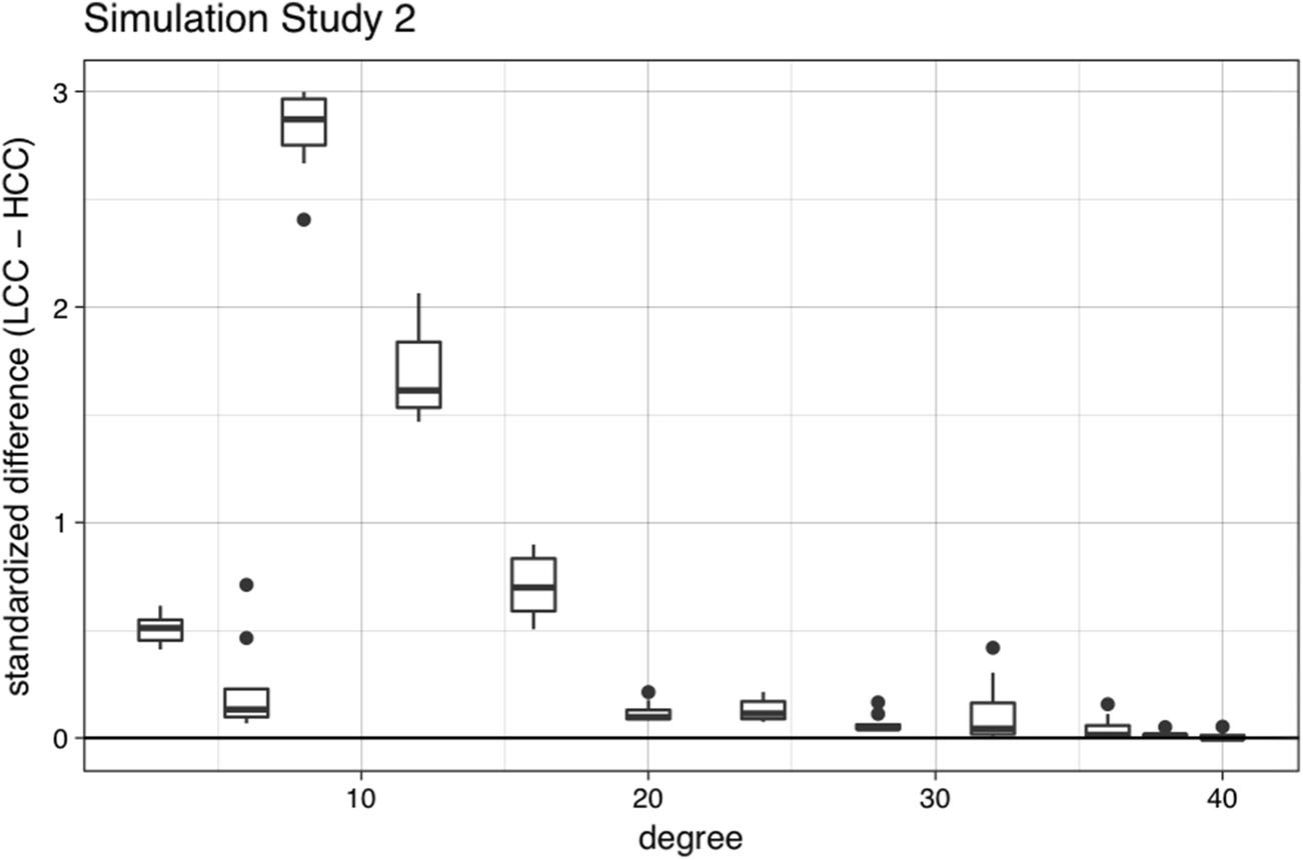
Mean standardized difference scores (with error bars) between the final activation of words with low clustering coefficients and words with high clustering coefficients with the same degree in Simulation Study 2. The difference score was obtained by subtracting the final activation of the high *C* word from the final activation of the low *C* word with the *same degree* and for the *same retention parameter* (i.e., for each value of degree, nine difference scores represented the nine different retention values [.1 to .9, in increments of .1]). The mean difference score was obtained by averaging across all retention values. In almost all cases, the final activation value of words with low clustering coefficients was greater than the final activation value of words with high clustering coefficients

Table 2 shows the standardized beta coefficients for each predictor and their corresponding *t* tests. The overall adjusted *R*^2^ was .683, *F*(3, 212) = 155.4, *p* < .001. Unsurprisingly, retention was a significant predictor of final activation values, such that higher retention rates were associated with higher final activation values. Clustering coefficient was also a significant predictor of final activation values, such that words with high clustering coefficients had lower final activations (corresponding to lower false alarm rates), replicating the behavioral findings in Experiment 1 of Vitevitch et al. (2012), who reported a higher false alarm rate for words with low clustering coefficients than for words with high clustering coefficients. Finally, degree was a significant predictor of final activation values, such that words with high degrees had lower final activations (corresponding to lower false alarm rates; see Fig. 7).

**Table 2:** Standardized beta coefficients for each predictor in the regression model in Simulation Study 2, and their corresponding *t* tests

	*β*	*SE*	*t*	*p*
Retention	0.77	0.116	6.68	< .001
Degree	– 2.21	0.120	– 18.48	< .001
Clustering coefficient	– 1.58	0.120	– 13.19	< .001

## Simulation Study 3: Semantic priming

The goal of Study 3 was to demonstrate how spreadr can be used to investigate the cognitive mechanisms that underlie semantic priming. Semantic priming is typically investigated via the lexical decision task, in which participants are presented with a prime followed by a target and have to decide, as quickly and accurately as possible, whether the target word was a real English word or a nonword. The general finding is that participants are faster and more accurate when the prime is related to the target (e.g., DOCTOR–nurse), as compared to when the prime is unrelated to the target (e.g., DOCTRINE–nurse; see Neely, 1991, and McNamara, 2005, for reviews of the semantic priming literature). The present simulations differed from the previous ones in two ways: (i) These simulations were conducted in a *semantic* network (instead of a phonological network), where edges were placed between words that were semantically related to each other, and (ii) the simulation outputs were compared against empirical data for the same prime–target pairs. The purpose was to provide a more stringent test of the capabilities of spreadr and to demonstrate how researchers can use spreadr to study cognitive processes in a different domain.

This set of simulations was conducted in a semantic network, in which edges were placed between words that represented the cues and responses in a free association task. In the free association task, a cue word is presented to participants who provide words that are related to the cue (e.g., listing the words “dog” and “kitten” in response to the cue word “cat”; De Deyne et al., 2018; Nelson, McEvoy, & Schreiber, 2004). Specifically, the semantic network used in the present set of simulations was constructed from the University of South Florida (USF) free association norms (Nelson et al., 2004) and was obtained from http://vlado.fmf.uni-lj.si/pub/networks/data/dic/fa/FreeAssoc.htm (where the cues and responses from the USF database were converted into a network representation in Pajek, a network analysis program). This Pajek network object was converted to an igraph network object for the present study, and directed and weighted edges in the Pajek network were converted to undirected and unweighted edges in the igraph network. Self-loops and duplicated edges were also removed, resulting in a network with 10,617 nodes and 63,782 edges.

The empirical data were obtained from the Semantic Priming Project (SPP; Hutchison et al., 2013; http://spp.montana.edu), a megastudy that collected speeded naming and visual lexical decision data for 1,661 words following related and unrelated primes from a large number of participants. For the present study, 100 targets were randomly selected from the set of 1,661 targets, and the related and unrelated primes associated with each target were retrieved (e.g., the target “ballet” with its related prime “tutu” and unrelated prime “officer”), resulting in 200 prime–target pairs. All 200 primes and 100 targets were included in the USF free association norms. The mean item *z*-scored lexical decision RTs (with a stimulus onset asynchrony of 1,200 ms) for each of the 200 prime–target pairs were then retrieved from http://spp.montana.edu. A list of the 200 prime–target pairs selected for the simulation is provided in Appendix 2.

The goal of the present study was to conduct a “virtual” experiment using the empirical data obtained from the SPP and the outputs of the simulations (i.e., final activation values of the target words in the semantic network) conducted with spreadr for the same set of prime–target pairs. If a process such as spreading activation implemented on a semantic network of free associations could be used to account for semantic-priming effects, one would expect to find higher activation levels of the target at the final time step to be correlated with faster RTs in lexical decision, and targets with related primes to have higher final activation levels than targets with unrelated primes. Although these hypotheses might appear to be trivial, it is important to demonstrate that spreading activation as implemented by spreadr in a semantic network representation is indeed able to account for the general semantic priming effect, especially given that spreading activation is generally accepted as the basic mechanism underlying semantic priming effects (McNamara & Altarriba, 1988; but see Lucas, 2000).

To investigate the ability of a simple spreading activation process to account for the advantage observed for related prime–target pairs, 100 units of activation were assigned to the *prime* at *t* = 0, and the spreading activation process was allowed to proceed for ten time steps. At the end of ten time steps, retrieval of the target was presumed to occur (as in Studies 1 and 2), and the final activation level of the *target* was recorded. The following parameters were used: four values of *retention* [.2, .4, .6, .8], to ensure that the results could be generalized across different amounts of retained activation; *suppress* = 0; and *decay* = 0. A total of 800 simulations were conducted (100 targets * 2 prime types * 4 values of retention).

### Results

The *z*-scored item mean RTs for the 200 prime–target pairs from the SPP were negatively correlated with the final activation levels of the target for all retention values [*r*_.2_ = – .264, *r*_.4_ = – .245, *r*_.6_ = – .262, *r*_.8_ = – .273; all *ps* < .001], indicating that targets that received more activation tended to be responded to more quickly in the lexical decision task. To examine whether prime type (related vs. unrelated to the target) was a significant predictor of the empirical data and simulation results, five linear regression models were fitted in which *z*-scored item mean RTs and the final activation level of the target for each retention value were the dependent variables. The key predictor of interest was prime type (i.e., related or unrelated to the target), and the following covariates were included: length of prime, frequency of prime, orthographic neighborhood size of prime, length of target, frequency of target, orthographic neighborhood size of target, forward association strength (the proportion of participants in Nelson et al. (2004), norms who reported the target in response to the prime), backward association strength (the proportion of participants in Nelson et al. (2004), norms who reported the prime in response to the target), CueFanOut (the number of targets given as a response to the prime when it was a cue in the Nelson et al. (2004), norms), TargetFanIn (the number of cues that produced the target as a response in the Nelson et al. (2004), norms), and semantic similarity computed via latent semantic analysis (LSA; Landauer & Dumais, 1997; this value represented the similarity between the prime and target based on their co-occurrences in text corpora). The values for these covariates were obtained from the SPP.

**Fig. 8:**
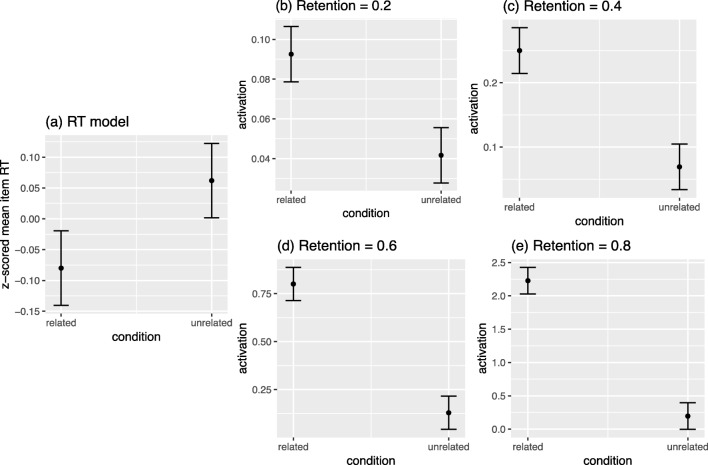
Marginal effects of prime type for all regression models. In all cases, targets that followed related primes were responded to more quickly (a) and had higher levels of activation in the spreadr simulations conducted (b–e), as compared to targets that followed unrelated primes

The results of the regression are shown in Table 3. After controlling for variables known to influence lexical decision performance (i.e., the lexical characteristics of the prime and target) and variables typically used to measure the relatedness of prime and target (Hutchison et al., 2013), targets that followed related primes corresponded with higher activation levels and were also responded to more quickly than targets that followed unrelated primes. Figure 8 shows the marginal effect of prime type, after controlling for covariates. Overall, the results of this “virtual” experiment showed that the simulations conducted using spreadr, which involved a simple process of spreading of activation implemented in a semantic network of free associations, was able to account for the semantic priming effect in lexical decision.

**Table3:**
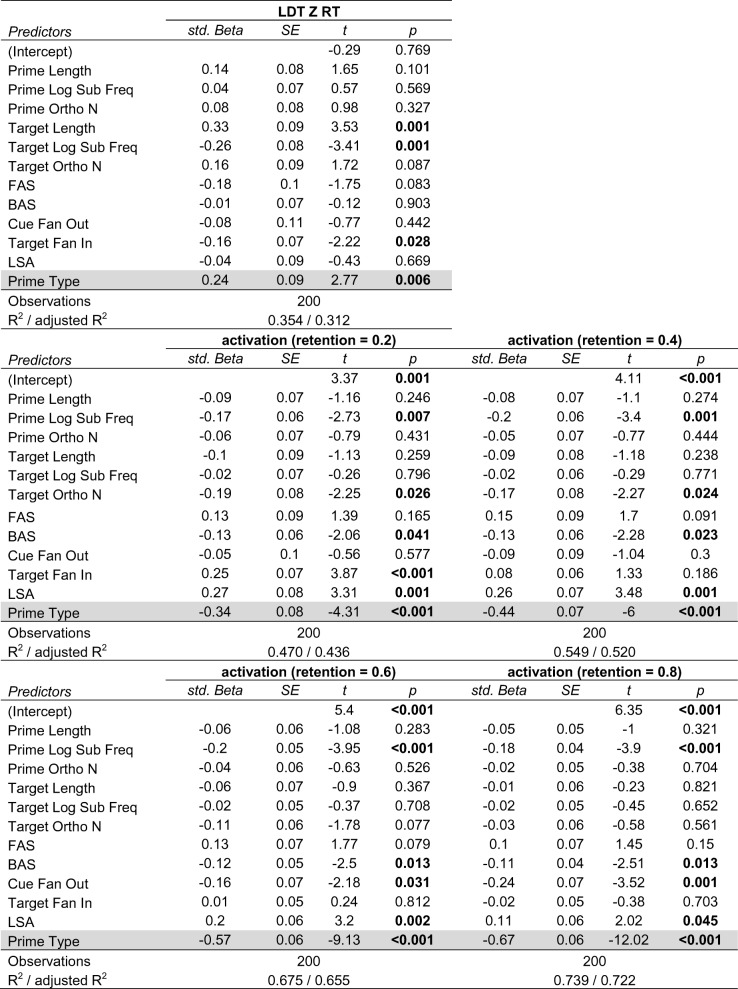
Standardized beta coefficients for each predictor in the regression models and their corresponding *t* tests. In all models, the “Prime Type” predictor was significant (highlighted in gray)

## General Discussion

This article introduced spreadr, an R package that can simulate spreading activation in a network of nodes and edges. Three sets of simulations demonstrated the utility of this tool to simulate spreading activation as a possible mechanism to account for the clustering coefficient effect in lexical retrieval and false memory, and for semantic priming effects in lexical decision. It fills a surprising gap in the literature—although the metaphor of spreading activation is very prevalent in cognitive psychology research (e.g., Anderson, 1983; Collins & Loftus, 1975), to the best of my knowledge, few tools are both freely available and accessible to psychologists that enable them to specifically test these ideas or intuitions computationally.

The simulation studies reported in this article demonstrate how the functions in spreadr can be readily extended to investigate a variety of cognitive phenomena. As was demonstrated in Study 3, spreadr can be used to examine how spreading activation occurs in a semantic network constructed from free associations (De Deyne et al., 2016; Nelson et al., 2004), and future work should examine whether various complex behavioral patterns of semantic priming (e.g., interactions with stimulus onset asynchrony, asymmetric priming effects, mediated priming effects; see McNamara, 2005, for a review) can be “reproduced” using spreadr, and how this implementation compares to a connectionist, neural network framework (e.g., Lerner, Bentin, & Shriki, 2012; Plaut & Booth, 2000). Indeed, a common and valid critique of spreading activation models is that they are “metaphorical models, which do not offer a mechanistic account of the dynamics in question” (Lerner et al., 2012, p. 3). With spreadr, however, cognitive scientists can begin to evaluate spreading activation models computationally, instead of merely discussing these models in metaphorical terms.

For instance, one research question in the semantic priming literature that could benefit from computer simulations conducted using spreadr involves the mediated semantic priming effect, which has a long history of research with contradictory results across different tasks (Balota & Duchek, 1989; Balota & Lorch, 1986; De Groot, 1983; de Wit & Kinoshita, 2015). One possible explanation for these findings is that researchers have failed to take into account the broader semantic structure of language when developing the stimuli (primes and targets) used in these experiments. For instance, consider the words *lion*–*tiger*–*stripes*. In two-step (mediated) priming, *lion* primes the target word *stripes*, mediated via the word *tiger*. However, it is conceivable that larger amounts of “long-range priming” might occur if there are multiple paths (through mediating words such as *tiger* and *zebra*) from the prime (*lion*) to the target (*stripes*), such that the target receives more activation accumulated from multiple sources (not an implausible idea, given past work showing that the accumulation of activation is additive in nature; Balota & Paul, 1996). A simulation of spreading activation among words in a semantic network for two or three steps and examining the probability distribution of activation values across all other words in the semantic lexicon could lead to new insights into mediated priming effects.

More importantly, the package and the simulations conducted exemplify a key idea in network science—that a complete understanding of any process should include a consideration of the structure in which the process is operating in. Such considerations may be particularly relevant to at least two diverse bodies of research: One related to the theoretical debate regarding models of retrieval from semantic memory, and one related to cognitive aging and decline.

First, a key theoretical debate in the area of semantic memory relates to the difficulty of disentangling the influences of structure and process in retrieval outputs from memory (i.e., responses in a category fluency task). For instance, Hills, Jones, and Todd (2012) found that a search process that dynamically switched between subcategories of a semantic space extracted from a text corpus could account for fluency data (see also Hills, Todd, & Jones, 2015). On the other hand, Abbott, Austerweil, and Griffiths (2015) argued that a random walk model operating on a semantic network of free associations is also a plausible mechanism of search in fluency tasks. The spreadr package could be useful to researchers who wish to conduct simulations to examine how information might be retrieved from a memory representation with varying structural properties (which could be approximated by a semantic network constructed of edges that represent free associations, shared features, or co-occurrences in text corpora), and could be extended to investigate how variations of the spreading activation process (i.e., adjusting the parameters of decay, suppress, retention) might interact with the structure of semantic memory to produce the outputs in the fluency task.

Second, spreadr could contribute to theoretical work related to cognitive aging and decline. As people age they accumulate more semantic information, resulting in denser semantic networks (Dubossarsky, De Deyne, & Hills, 2017; Ramscar, Hendrix, Shaoul, Milin, & Baayen, 2014; Wulff, Hills, Lachman, & Mata, 2016). Together with research showing that older adults experience declines in episodic memory (Balota, Dolan, & Duchek, 2000; Naveh-Benjamin, Hussain, Guez, & Bar-On, 2003) and more frequent lexical search and retrieval difficulties (Burke, MacKay, Worthley, & Wade, 1991; James & Burke, 2000), an important theoretical question is the extent to which the cognitive deficits observed in older adults are due to a denser semantic structure or due to a process that has become less “efficient.” Using the spreadr package, one could (i) compare spreading activation in sparse and densely connected network structures, and (ii) manipulate the parameters (i.e., decay, retention, suppression) to simulate an efficient or inefficient process. For instance, specifying a higher decay rate, higher retention rate, or higher suppression threshold would introduce “friction” into the spreading activation process, so that activation would not spread as easily in the network. Therefore, spreadr provides a computational “sandbox” for researchers to explore (albeit on a small scale) the interaction between structure and process.

In closing, it must be emphasized that the intention of this article was not to provide a definitive theory or model of spreading activation; rather, its purpose is to enable the broader application of spreading activation in a specified network structure, which could represent a language network or semantic network or any cognitive network of interest. The approach used here has relatively few parameters as compared to more established and prominent models, such as the interactive-activation model (McClelland & Rumelhart, 1981) and connectionist models (Gordon & Dell, 2001; Harm & Seidenberg, 2004; Seidenberg & McClelland, 1989), that are more complex and have several parameters that must be carefully tuned to improve performance. The approach here complements these models, but differs from them by focusing on exploring a single, simple idea—how spreading activation might occur in a network of connected nodes. As was noted by McClelland (2009), cognitive models were never intended to fully account for any cognitive phenomenon, but rather are “explorations of ideas about the nature of cognitive processes” (p. 11). Conducting simple simulations allows us to clearly test specific ideas related to cognitive processing.

There is much value in computationally testing verbal theories, and such research can complement behavioral and experimental approaches in cognitive psychology research (Lewandowsky, 1993; Farrell & Lewandowsky, 2010). It is hoped that this package will be useful to cognitive and language scientists who are interested in investigating spreading activation in a concrete way, and will encourage others to consider how the structure of cognitive systems such as the mental lexicon and semantic memory interacts with the process of spreading activation.

## Figures and Tables

**Fig. 9:**
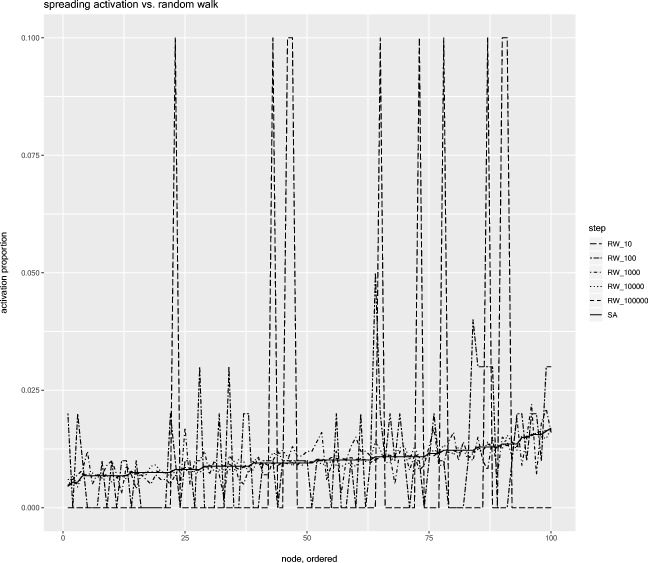
Proportion of activation for each node from the SA simulation (solid line) and the random walk probabilities for each node based on 10, 100, 1,000, 10,000, and 100,000 walks (dashed or dotted lines). The outputs begin to converge when more random walks are implemented

**Fig. 10:**
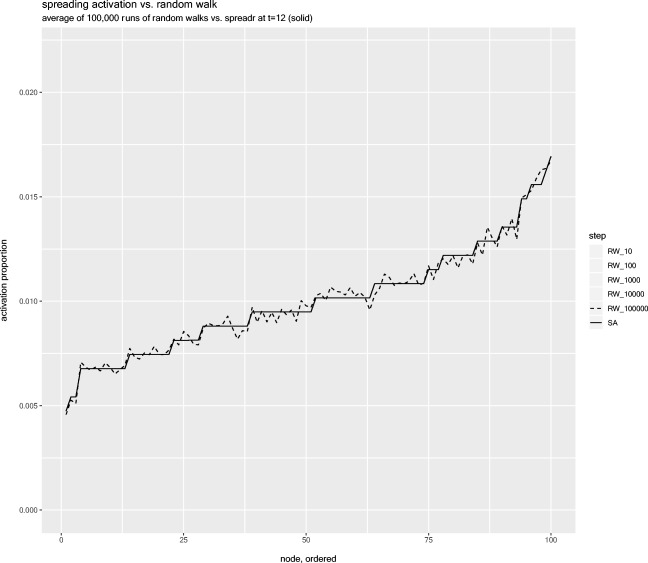
Proportion of activation for each node from the SA simulation (solid line) and the random walk probability for each node based on 100,000 walks (dotted line)

## Appendix 1

**Table 4.** Degree and clustering coefficient values of the 24 target nodes used in Simulation Studies 1 and 2

Node	Degree	Clustering Coefficient
1	3	.67
2	3	.33
3	6	.47
4	6	.13
5	8	.46
6	8	.14
7	12	.47
8	12	.14
9	16	.49
10	16	.16
11	20	.61
12	20	.27
13	24	.59
14	24	.26
15	28	.46
16	28	.24
17	32	.39
18	32	.21
19	36	.32
20	36	.21
21	38	.28
22	38	.20
23	40	.31
24	40	.23

## Appendix 2

**Table 5.** List of prime–target pairs randomly selected from the Semantic Priming Project (Hutchison et al., 2013) for Simulation Study 3

Target	Related Prime	Unrelated Prime
africa	continent	straight
ambulance	emergency	cape
baby	child	inside
ballet	tutu	officer
bass	treble	meticulous
begin	originate	professional
blow	whistle	acid
bother	nag	perceive
breathe	air	hay
building	architect	what
camera	tourist	excuse
climb	stairs	chisel
coat	trench	vegetable
compact	disc	criterion
cool	refreshing	precipice
cough	sneeze	drive
crack	crevice	annoy
dancer	singer	kettle
dead	corpse	mineral
defeat	conquer	slug
dinner	lunch	join
dolphin	flipper	sheet
doughnut	pastry	ill
east	west	stewardess
exhale	inhale	gravy
explain	elaborate	tar
far	distance	monument
fashion	fad	command
feet	hands	spell
finish	accomplish	god
five	four	wagon
gas	fuel	unique
general	specific	soon
gin	tonic	resort
give	generous	bumps
glory	hope	influence
glue	paste	moss
gone	going	federal
grasp	hold	spread
guilty	innocence	bug
have	must	newspaper
hill	steep	birthday
hold	grip	equal
human	being	catch
indian	tribe	breakable
insurance	claim	wheel
itch	rash	boots
item	product	let
juvenile	delinquent	jury
know	intuition	wool
lawyer	attorney	central
leg	crutch	appendage
live	survive	virgin
many	variety	owe
more	extra	convict
move	shift	oasis
nephew	niece	captain
obsession	compulsion	interesting
paint	art	some
paper	news	inmate
pass	fail	motel
pen	quill	freckle
persuade	convince	rebel
planet	venus	author
plug	outlet	map
prejudice	stereotype	innocent
present	gift	jerk
priest	monk	close
protect	defend	bowl
refuse	denial	comma
remember	reminiscence	cougar
rent	lease	up
ride	bike	guilt
rule	policy	memory
run	jog	thin
sample	example	tax
science	chemistry	move
service	memorial	buzz
shake	malt	sibling
shame	disgrace	thimble
shave	foam	macaroni
sheep	lamb	entry
shopping	spree	pumpkin
smoke	cigar	lane
smooth	rough	relax
sorority	fraternity	slave
spine	backbone	real
spoiled	brat	argument
stamp	postage	flood
state	governor	cotton
stream	brook	annual
student	graduate	spatula
tall	grow	frog
thief	crook	blueberry
tire	flat	sneakers
toes	feet	mate
trees	landscape	fashion
values	morals	sleeve
wolf	coyote	coal
worm	maggot	anarchy

## Appendix 3 Results of simulations comparing the outputs of spreadr to naïve random walks implemented in the same network

All simulations were conducted on the same Erdös–Renyí random network of 100 nodes and 738 unweighted, undirected edges (~ .15 probability of an edge existing between two nodes).

Parameters used in the spreading activation simulation (SA) implemented by spreadr: 100 units assigned to a single node at *t* = 0; decay = 0, retention = 0, suppress = 0. The simulation was allowed to continue until the activation levels of all nodes were stable (i.e., changed by less than 0.001 units of activation). The simulation continued for 12 time steps based on this criterion. Time taken: 0.007 s.

Random walk simulations (RW), implemented by the *random_walk* function from the igraph R library: 100,000 random walks of 12 steps (analogous to the 12 time steps in SA) originating from the same node as above. Time taken: ~ 1.4 h.

### Results

The final activation levels of all nodes from the SA simulation were divided by 100 units in order to obtain the proportion of activation that “ended up” at each node at the end of 12 time steps.

The probability that the random walk “ended” at each of the 100 nodes was computed from the outputs of 10, 100, 1,000, 10,000, and 100,000 walks. The results can be seen in Figs. 9 and 10 below.

Overall, the outputs from the random walk model converge to the outputs from spreadr, but only when the random walk is repeated several times (at least 10,000 times). Hence, the outputs from spreadr could be argued to reflect the long-run behavior of the naïve random walker (i.e., when an infinite number of random walks have been taken), but computed with a fraction of the time it takes to complete a large number of random walks (less than 1 s, as compared to 1 h). It is important to emphasize that the two cases converge when the parameters of spreadr are set to specific values (i.e., decay = 0, retention = 0, suppress = 0). Additional work will be required to determine whether the two models converge when different parameter values are used for the spreading activation simulation.

**Table 6.** Correlations between the SA and RW outputs for various conditions

	*r*	*p*
SA | RW_10	.137	.173
SA | RW_100	.342	**.005**
SA | RW_1000	.616	**< .001**
SA | RW_10000	.927	**< .001**
SA | RW_100000	.993	**< .001**
